# Knowledge, Responsibilities, and Peer Advice From Care Partners of Patients With Parkinson Disease Psychosis

**DOI:** 10.3389/fneur.2021.633645

**Published:** 2021-02-01

**Authors:** Sneha Mantri, Briana Edison, Lamees Alzyoud, Steven M. Albert, Margaret Daeschler, Catherine Kopil, Connie Marras, Lana M. Chahine

**Affiliations:** ^1^Department of Neurology, Duke University, Durham, NC, United States; ^2^Department of Neurology, University of Pittsburgh, Pittsburgh, PA, United States; ^3^Department of Behavioral and Community Sciences, University of Pittsburgh, Pittsburgh, PA, United States; ^4^The Michael J Fox Foundation for Parkinson's Research, New York, NY, United States; ^5^The Edmond J Safra Program in Parkinson's Disease, Toronto Western Hospital, University of Toronto, Toronto, ON, Canada

**Keywords:** parkinson disease, care partners, psychosis experiences, peer advice, mixed-methods analyses

## Abstract

**Introduction:** Care partners (CPs) of individuals with Parkinson disease psychosis (PDP) experience increased strain and rely on informal support networks. The objective of this study was to characterize CP responsibilities, sources of support, and peer advice.

**Methods:** This was a mixed-methods cross-sectional study. The sample was recruited from the online Fox Insight study cohort. CPs who indicated their care recipient suffered hallucinations and/or delusions were administered a questionnaire regarding their caregiving experience to person with PDP. A free-text question asked CPs to give advice to a hypothetical peer CP. Responses to multiple-choice questions were tabulated; responses to the free-text question were grouped into advice categories.

**Results:** 145 CP of individuals with PDP were included in this analysis, mean age (standard deviation, SD) 66.4 (9.4) years; 110 (75.9%) were women. Most (115, 79.3%) provided caregiving on a daily basis, with a range of responsibilities. Only 16 (11%) learned about PDP from a physician; communication challenges included perceived embarrassment or having to prioritize other issues in a limited appointment time. The most common peer advice was to alert the care recipient's neurologist (*n* = 38, 30.4%); only 8 (6.4%) suggested medication changes.

**Conclusion:** CPs face challenges with clinician communication and learn about psychosis from a variety of informal sources. Few CPs advocate for medications to control PDP, instead preferring non-pharmacological management strategies. Peer advice favored alerting the care recipient's physician, suggesting that CPs do desire more information from the medical team.

## Introduction

Psychosis affects up to 80% of individuals with advanced Parkinson disease (PD) (1), impacting not only the patient but also their family members. Presence of PD psychosis (PDP) is associated with impairment in daily activities (2) and may necessitate increasing caregiving requirements. Individuals who provide regular care for patients with PDP, or “care partners,” (CPs) provide large amounts of uncompensated care and experience higher levels of psychosocial strain (3). Additionally, caregiver burden is independently correlated with institutionalization of the person with PD (4) and with worsened mental and physical health of CPs. Thus, strategies to reduce caregiver burden in this population are needed in the care of people with PD (5).

While there are few controlled studies of such strategies for reducing the burden of CP for patients with PDP, two general approaches have been advocated (6): treatment of the psychosis symptoms in the care recipient, and provision of education to the CP. Treatment of psychosis in PD remains challenging, with few evidence-based, clinically efficacious therapies (7). In addition to adjustment of PD medications and removal of other inciting agents, pharmacotherapy remains the mainstay of treatment for PDP, but use of anti-psychotic medications in PD is associated with substantial increased risk of mortality, even after adjusting for measurable confounders (8). Decisions to initiate treatment may be guided by the severity and distress of the PDP in the patient. A better understanding of caregiver attitudes regarding pharmacotherapy for PD psychosis could help the shared decision to initiate such therapy for PDP.

As for education of CP as a means of reducing burden, incorporating CP preferences into the modality, materials, and sources preferred could improve CP satisfaction with and benefit from such materials. One potential strategy to reduce CP burden is creation of peer support materials. Peer support programs have demonstrated benefit for a number of other mental health conditions (9). Family peer support networks have also been beneficial for schizophrenia (10) as well as neurodegenerative conditions such as amyotrophic lateral sclerosis (11). Many such programs provide advice “from families to families” in an attempt to provide CPs with actionable strategies to reduce symptom burden or improve communication between the individual with the illness, their CP or family member, and medical professionals.

We aimed, broadly, to study these 2 aspects related to reducing CP burden from PDP. Specifically, our objectives were to (1) determine attitudes of CP of PDP toward medications for psychosis and experiences with communicating with healthcare providers about medications (2) ascertain sources of education for CP about PDP, and (3) explore the peer advice that CP offer, to inform developing of support materials specific to CPs of people with PDP.

## Methods

### Design

This was a mixed-methods cross-sectional survey study with an embedded parallel design. Subjects were recruited through the Fox Insight (FI) study. Fox Insight study methods have been described elsewhere in detail (12). Briefly, FI is an online-only longitudinal observational study in which individuals with and without self-reported PD participate in study activities via an online platform. For this study, individuals participating in FI who had identified themselves as being caregivers for individuals with PD were eligible to participate and received an email invitation from the study team in September 2019. The email stated “The current research explores how caregivers experience and communicate about their life of caring for someone with Parkinson's disease. We also explore the effect psychiatric symptoms in Parkinson's disease have on the caregiver experience. In this Fox Insight survey, we seek to better understand these topics.” A subset of individuals who were not directly invited by the study team, but who received the email invitation via snowball sampling, also could participate. Recipients of the email could then click on a link within the email that took them to the survey.

### Survey Questions

The survey consisted of a series of questions regarding caregiving time commitments and roles, as well as the Neuropsychiatric Inventory Questionnaire (NPI-Q) and Caregiver Burden Index (CBI). Those who reported, on the NPI-Q, hallucinations/delusions in their care recipient with PD were administered additional multiple-choice questions regarding medication use, experiences with physician communication regarding PDP, sources of knowledge about PDP, and preferred sources of such knowledge, as follows:

- Does the person you care for take any of the following medication(s)? Please select all that apply:
  - Pimavanserin (Nuplazid), Quetiapine (Seroquel), Risperidone (Risperdal), Olanzapine (Zyprexa), Clozapine (Clozaril), Haloperidol (Haldol), Donepezil (Aricept), Rivastagimine (Exelon), Memantine (Namenda), Clonazepam (Klonopin), Lorazepam (Ativan), None of the above, I do not know the medication(s) they currently take
- What prompted your [care recipient's] health care provider to prescribe medication to treat their psychosis symptoms? Please select all that apply.
  - They became a danger to themselves
  - They became a danger to others
  - As a caregiver, I could no longer provide adequate care due to their psychosis symptoms
  - Their psychosis symptoms were scaring or otherwise negatively impacting me as a caregiver
  - The psychosis symptoms were scaring or otherwise negatively impacting my
  - Some other reason, specify.
- How did you first learn about Parkinson's disease psychosis? (select all that apply)
  - The doctor of the patient I care for with Parkinson's disease informed me about psychosis
  - When the person I care for started experiencing psychosis symptoms
  - Someone at a support group informed me
  - From a PD organization such as Parkinson's Foundation, MJFF, APDA
  - Research I did myself online or through books
  - From a television commercial
  - Other, specify.
- What information, if any, would have been most helpful to have when the person you care for with Parkinson's disease started experiencing psychosis symptoms? Please select up to three.
  - Information on the causes of psychosis
  - Information on the treatments for psychosis in PD
  - Information on medication to treat psychosis symptoms
  - Information on support groups
  - Information on how best to handle the person you care for when they experience a psychosis episode
  - Information explaining the symptoms of psychosis (i.e., hallucinations, delusions, and paranoia)
  - Information on books and/or articles to read about psychosis symptoms
  - Some other type of information (Please specify in the box below)
  - None
- Peer advice: the last question in the psychosis sub-survey was open-ended: “What is one piece of advice would you give to a caregiver when the person they care for first starts experiencing psychosis symptoms (i.e., hallucinations, delusions and/or paranoia)?”

These questions were developed from qualitative analysis of CP journals and semi-structured interviews (13) in order to ensure that areas of importance for CPs were included.

### Analysis

For this analysis, subjects were included if they had available information on age, sex, and had completed the NPI-Q items on hallucinations and delusions. Responses to multiple choice questions (i, ii, iii, iv above) were summarized with descriptive statistics; responses to the free-text question on peer advice were analyzed by the study team [BE, SM, LC] and grouped into categories; responses could be coded into multiple categories. A codebook was developed by the primary coder [BE] and reviewed for accuracy by two movement disorders specialists with experience treating PDP[SM and LC]. Sex differences between male and female CPs were assessed using the chi-square test.

The multiple-choice question responses analyzed in this study are openly available in FoxDen at https://foxden.michaeljfox.org/insight/explore/insight.jsp. This study was performed in accordance with the Declaration of Helsinki. This study is approved by the New England Institutional Review Board, and informed consent is obtained from each participant at enrollment.

## Results

Two thousand seven hundred forty individuals received the survey email (2433 invited through fox insight, 302 recruited via snowball sampling). Of these, 741 clicked on the link to open the survey (response rate 27%). Compared to those who did not open the survey, those who did were older (64.9 vs. 62.5 years, *p* < 0.001) and more likely to be male (29.4% male vs. 20.6% male, *p* < 0.001).

The NPI-Q was completed by 471 respondents of whom 145 (30.8%) endorsed hallucinations (*n* = 126) or delusions (*n* = 64) in the care recipient with PD; 45 endorsed both hallucinations and delusions. CPs of patients with hallucinations and/or delusions had higher NPI-Q total score (mean (standard deviation, SD) 10.72 (5.99) vs. 4.37 (3.96) respectively, *p* < 0.001) and CBI scores (mean (SD) 41.83 (15.80) vs. 26.88 (16.72), respectively, *p* < 0.001) compared to those without either.

Among CPs of patients with hallucinations and/or psychosis, mean age (standard deviation, SD) was 66.4 (9.4) years, and 110 (75.9%) were women; 96 (66.2%) were not employed outside the home. The mean (SD) duration of disease in the care recipient was 7.5 (7.0) years. The relationship between the CP and the care recipient with PD, as well as time spent on caregiving activities, is shown in Table 1. Most CPs reported spending time in the caregiving role on a daily basis (*n* = 115, 79.3%), with a combination of physical, emotional, medical, and financial responsibilities (Table 1). CPs attributed a variety of physical and emotional symptoms in themselves to their caregiving role, most notably exhaustion (*n* = 106, 73.1%) and depression/sadness (*n* = 64, 53.8%). Male CPs were more likely to report exhaustion than female CPs; no other sex differences were noted. Favored coping strategies included respite care (*n* = 98, 67.6%) and exercising (*n* = 79, 54.5%).

**Table 1:** Demographics of respondents, caregiving roles, and symptoms attributed to caregiving (*N* = 145).

**Relationship between care partner and care recipient**	***n* (%)**	**Male (*n* = 35)**	**Female (*n* = 110)**	***p***
Spouse/partner	106 (73.1)	30 (85.7)	76 (69.1)	n.s.
Parent-child	26 (17.9)	2 (5.7)	24 (21.8)	0.044
Sibling	6 (4.1)	0 (0)	6 (5.5)	n.s
Aunt/uncle-niece/nephew	0 (0)	0 (0)	0 (0)	n.s
Employee	0 (0)	0 (0)	0 (0)	n.s
Other	7 (4.8)	3 (8.6)	4 (3.6)	n.s
**Time spent caregiving**	***n*** **(%)**			
Full time	85 (58.6)	26 (74.3)	59 (53.6)	n.s
Few hours daily	30 (20.7)	7 (20)	23 (20.9)	n.s
Few days weekly	18 (12.4)	2 (5.7)	16 (14.5)	n.s
One day during the week	12 (8.3)	0 (0)	12 (10.9)	n.s.
**Responsibilities as caregiver**	***n*** **(%)**			
Emotional support	133 (91.7)	34 (97.1)	99 (90)	n.s.
Transportation	126 (86.9)	34 (97.1)	92 (83.6)	0.039
Handling crisis or medical emergency	123 (84.8)	33 (94.3)	90 (81.8)	n.s.
General health care	122 (84.1)	33 (94.3)	89 (80.9)	n.s.
Food preparation	116 (80.0)	31 (88.6)	85 (77.2)	n.s.
Home organization	116 (80.0)	31 (88.6)	85 (77.2)	n.s.
Financial	115 (79.3)	34 (97.1)	81 (73.6)	0.003
Obtaining/administering medications	115 (79.3)	32 (91.4)	83 (75.5)	0.042
Mobility assistance	99 (68.3)	32 (91.4)	67 (60.9)	0.001
Assisting with personal care	97 (66.9)	31 (88.6)	66 (60)	0.002
Other	30 (20.7)	11 (31.4)	19 (17.3)	n.s.
**Symptoms attributed to caregiving role**	***n*** **(%)**			
Exhaustion	106 (73.1)	32 (91.4)	74 (67.3)	0.005
Difficulty sleeping	93 (64.1)	25 (71.4)	68 (61.8)	n.s.
Depression/sadness	78 (53.8)	21 (60)	54 (49.0)	n.s.
Anxiety	70 (48.3)	14 (40)	56 (50.9)	n.s.
Feeling overwhelmed	65 (44.8)	14 (40)	51 (46.4)	n.s.
Frustration	64 (44.1)	15 (42.9)	49 (44.5)	n.s.
Change in weight	51 (35.2)	14 (40)	37 (33.6)	n.s.
Physical pain	45 (31.0)	16 (45.7)	33 (30)	n.s.
Fear	42 (29.0)	11 (31.4)	31 (28.2)	n.s.
Headaches	38 (26.2)	9 (25.7)	29 (29)	n.s.
Being uncomfortable	33 (22.8)	10 (28.6)	23(20.9)	n.s.
Hair loss	17 (11.7)	4 (11.4)	13 (11.8)	n.s.
Embarrassment	15 (10.3)	2 (5.7)	13 (11.8)	n.s.
Other physical symptom	33 (22.2)	11 (31.4)	22 (20)	n.s.
Other emotional symptom	16 (11.0)	6 (17.1)	10 (9.1)	n.s.
No symptoms	6 (4.4)	0 (0)	6 (5.5)	n.s.

*Percentages may add to more than 100% as respondents could select multiple answers*.

CPs learned about psychosis from a variety of sources, including personal research (*n* = 31, 21.4%) and from disease-specific organizations (*n* = 23, 15.9%). A minority (*n* = 16, 11.0%) learned about psychosis from a physician. Several challenges were noted in communicating with a care recipient's physicians, including not wanting to speak about PDP in front of a patient (*n* = 21, 14.4%), not wanting to embarrass the patient (*n* = 25, 17.2%) and feeling there were more important symptoms to discuss during appointments (*n* = 27, 18.6%). CPs expressed a desire for more information regarding PDP, including practical advice on how to handle PDP (*n* = 99, 68.3%), explanation of PDP (*n* = 68, 46.9%), and options for treatment (*n* = 56, 38.6%).

Forty-six respondents (31.7%) reported that their care recipient was taking an antipsychotic medication; of these, 35 (76.1%) were taking an acetylcholinesterase inhibitor, 33 (71.7%) were taking a benzodiazepine, and 28 (60.9%) were taking a D2 antagonist. The three most common reasons CPs chose to advocate for a medication (hypothetically or in reality) were perceived danger to the patient (*n* = 45, 31.0%) or the CP (*n* = 31, 21.4%), or if PDP posed a barrier to other care (*n* = 35, 24.1%). One CP indicated that they would not advocate for antipsychotic medications in any situation.

Of the 145 CPs included in the sample, 124 provided free-text advice to hypothetical peers. Mean response length was 12.6 words (SD 10.9). A detailed codebook with representative quotations can be found in Table 2. The most common piece of advice was to alert the care recipient's neurologist (*n* = 38, 30.4%) (“make the doctor aware immediately,” “stay in contact with specialists”); other frequent themes were to remain calm (*n* = 34, 27.2%; “be calm and patient with the person”), to support the patient (*n* = 29, 23.2%) (“Treat them with care and respect- always be calm and loving”), and to reassure the patient (*n* = 19, 15.2%) (“Constantly reassure the patient that all is well”). Advice categories also included being patient, positive, and obtain resources and education. Unfortunately, some respondents expressed helplessness at the situation. Only eight CPs (6.4%) suggested medication adjustments as a potential solution to PDP; two of these specifically named pimavanserin. No other specific medications were named in any of the responses. There were no sex differences in the types of free text responses provided.

**Table 2:** Codebook for peer advice regarding psychosis (*N* = 124).

**Category**	**Definition**	**Sample text**	***n* (%)**	**Male**	**Female**	***p***
				***N* = 34**	***N* = 90**	
Talk to doctor	Advising to alert, discuss with, or seek advice from doctor/neurologist	Make the doctor aware immediately. Stay in contact with specialists.	38 (30.4)	9 (26.5)	29 (32.2)	n.s.
Positivity	Alluding to keeping a positive attitude and light mood	I try to laugh about it. My husband is not frightened by what he sees so I make light of it.	3 (2.4)	1 (2.9)	2 (2.2)	n.s.
Safety	Taking safety precautions and being prepared for any harm or injury	Watch them carefully to ensure they do not harm anyone/thing/themselves.	4 (3.2)	1 (2.9)	3 (3.3)	n.s.
Support patient	Attempting to keep patient calm by caring for, listening to, and discussing with them	Be gentle with the person. Offer to inject yourself into the situation (like checking if there is someone outside, or shooing the animal away).	29 (23.2)	10 (29.4)	19 (21.1)	n.s.
Remain calm	Suggestions for caregiver to remain even-tempered, not heighten or escalate situation by fighting, arguing, or judging	Be calm and patient with the person, do not confront about being “wrong” about the experience, it is right to the person in that moment.	34 (27.2)	9 (26.5)	25 (27.8)	n.s.
Reassurance/ rationalize	Explaining away the situation and reassuring all is okay	Constantly reassure the patient that all is well.	19 (15.2)	6 (17.6)	13 (14.4)	n.s.
Distraction	Changing setting, activity, or topic to divert attention	Redirect their behavior as you would for a young child.	10 (8)	3 (8.8)	7 (7.8)	n.s.
Educate/resources	Using educational tools and resources provided by PD experts and other caregivers (i.e., support groups)	Read many resources so when “it” happens, you are not surprised and may be able to better help.	12 (9.6)	3 (8.8)	9 (10)	n.s.
Medication	Introduce use of medication, seek information regarding medication, or adjustment of medication	See if they are able to take one of the drugs to help. Consider medication choice carefully.	8 (6.4)	2 (5.9)	6 (6.7)	n.s.
Patience	Exhibiting patience with PD patient	Be patient and understanding and be willing to listen.	11 (8.8)	2 (5.9)	9 (10)	n.s.
Acceptance	Accept the symptoms and realize it is part of the disease	Remember that this person has a brain injured from PD and cannot change the problem.	9 (7.2)	1 (2.9)	8 (8.9)	n.s.
Acknowledge	Recognize that symptoms are present and can be managed; make note of symptoms	Listen to what they are saying and acknowledge their fear.	13 (10.4)	5 (14.7)	8 (8.9)	n.s.
I don't know	Not sure, I don't know, none	I have no solutions/advice to give. Nothing worked for me.	7 (5.6)	2 (5.9)	5 (5.6)	n.s.

## Discussion

Reducing CP burden in PD is important to the health outcomes and quality of life in both persons with PDP and their CP. Pharmacotherapy for PDP, and provision of education to CPs, are two strategies that have been proposed to achieve this. Understanding CP's attitudes toward medications, and preferred sources of education, is needed to optimize delivery of these interventions. In addition, incorporation of peer advice into educational materials for CPs may be of benefit.

Consistent with other studies examining caregiving in advanced disease, CPs in our cohort were mostly female spouses of patients and provided a significant portion of unpaid or underpaid care (14, 15). They endorse various adverse physical and emotional consequences of their caregiving role, highlighting the importance of interventions to reduce burden among CP.

CPs of individuals with PDP report a variety of challenges obtaining information and treatment for their loved ones. They express a strong desire for information and anticipatory guidance regarding PDP and indicated that they obtained most information from non-physician sources. This reinforces the important role that formal and informal social and support networks may have for individuals with neurologic disorders and their families, including support groups, patient advocacy groups, and foundations (16).

Interestingly, the most common free-text peer advice category was to alert the care recipient's neurologist. However, few CPs suggested medication adjustment and some specifically advised their peers to turn to medications last, indicating that CPs turn to and desire more non-pharmacological input from a professional care team in navigating this stage of the disease. This is in contrast to the medication-oriented approach often favored by clinicians. As PDP is often a driver of CP burden and care recipient institutionalization (4, 17), this disconnect between physician and CP highlights a major unmet need in the PD community.

Noted strengths of the current study are a large sample from a geographically diverse cohort of CPs of people with PD, which allowed for characterization of a wide range of CP responsibilities and challenges, as well as the development of a detailed and comprehensive codebook. Nevertheless, some important limitations should be noted. First, the self-reported nature of Fox Insight limits our ability to confirm the diagnosis in the care recipient. The pattern of responses from the online Fox Insight cohort is similar to well-defined PD cohorts assessed in person (18) and agreement between self-reported diagnosis and clinician virtual visit is high (19). However, we cannot rule out the possibility that some of the CPs were caring for individuals with parkinsonism due to disorders besides PD (i.e., atypical parkinsonian syndromes), which could have increased caregiving needs. The present study was cross-sectional in design, but the longitudinal nature of the Fox Insight cohort could allow for repeated assessment over time. The majority of respondents were women, which is consistent with the clinical prevalence of women as CPs but may not adequately reflect the experiences of male CPs (20). Intriguingly, male CPs were more likely than female CPs to report specific responsibilities around medications, personal care, finances, transportation, and mobility, and male CPs were more likely to report feeling exhausted from caregiving. This is consistent with other work suggesting that male and female CPs view their roles differently (20) and may indicate that support structures for male and female CPs will need to be tailored to differing roles and responsibilities.

Although the lifetime prevalence of PDP approaches 80% (1), fewer than one in three survey respondents indicated hallucinations and/or delusions in their care recipient. This could indicate that care recipients are at a relatively early stage in the disease, as psychosis tends to manifest later in the course of the disease (1); CPs may also be unaware of some psychosis in the care recipient. Alternatively, there may be true differences in disease spectrum and severity between Fox Insight and other disease cohorts, although given the high concordance between Fox Insight and in-person assessments (18, 19) this seems less likely. However, those experiencing PDP, or their CPs, may be less inclined or able to sign up for and complete a series of online surveys. Additionally, the multiple-choice nature of an online survey limits respondents to select from a pre-defined group of choices. We attempted to mitigate this by randomizing the order in which choices were presented and including free-text options wherever feasible. As part of the PDEC, we also conducted semi-structured interviews with CPs to allow for more open-ended discussion of clinical approaches to managing PDP, including medication adjustments or screening for superimposed causes of delirium (13), which forms an important complementary qualitative analysis to the present work. Lastly, questionnaires such as the NPI-Q may fail to capture the range of responses that could develop from a nuanced clinician interview. Newer self- and caregiver-reported outcome measures, such as the PsycH-Q (21) may be both feasible and clinically appropriate to implement alongside traditional clinician-assessed metrics.

The results of this study can inform development of materials aimed at physicians, other health professionals, and CPs caring for individuals with PDP. For example, future studies could test various print/online formats in order to determine the best way of disseminating peer advice among CPs. Although some resources exist on disease-foundation and academic websites, wider dissemination is needed. A task force of key stakeholders, ranging from CPs to clinicians, would be suited to determining the best content and format for supporting CPs of those with PDP. Addressing CP concerns around this challenging stage of disease could potentially reduce caregiver burden and associated health care costs, improve the CP/recipient dynamic, and improve the overall quality of care for individuals living with Parkinson disease.

## Data Availability Statement

The data that support the findings of this study are openly available in FoxDen at https://foxden.michaeljfox.org/insight/explore/insight.jsp.

## Ethics Statement

The studies involving human participants were reviewed and approved by New England Institutional Review Board. The patients/participants provided their written informed consent to participate in this study.

## Author Contributions

SM developed the methodology, analyzed the data, wrote the original draft, reviewed the manuscript for critical content, and revised the manuscript. BE analyzed the data, created the figure, and reviewed the manuscript for critical content. LA analyzed the data, created the tables, and reviewed the manuscript for critical content. SA validated the analysis and reviewed the manuscript for critical content. MD administered the project, curated the data, and reviewed the manuscript for critical content. CK conceptualized the project and reviewed the manuscript for critical content. CM conceptualized the project, supervised the study team, and reviewed the manuscript for critical content. LC conceptualized the project, analyzed the data, supervised the study team, and reviewed the manuscript for critical content. All authors contributed to the article and approved the submitted version.

## Conflict of Interest

The authors declare that the research was conducted in the absence of any commercial or financial relationships that could be construed as a potential conflict of interest.

## References

[1] Gibson G Mottram PG Burn DJ Hindle JV Landau S Samuel M. Frequency, prevalence, incidence and risk factors associated with visual hallucinations in a sample of patients with Parkinson's disease: a longitudinal 4-year study. Int J Geriatr Psychiatr. (2013) 28:626–31. 10.1002/gps.3869 22927195

[2] Aarsland D Larsen JP Cummings JL Laake K. Prevalence and clinical correlates of psychotic symptoms in parkinson disease: a community-based study. Arch Neurol. (1999) 56:595–601. 10.1001/archneur.56.5.595 10328255

[3] Martinez-Martin P Rodriguez-Blazquez C Forjaz MJ Frades-Payo B Agüera-Ortiz L Weintraub D. Neuropsychiatric symptoms and caregiver's burden in Parkinson's disease. Parkinsonism Relat. Disord. (2015) 21:629–34. 10.1016/j.parkreldis.2015.03.024 25892660

[4] Abendroth M Lutz BJ Young ME. Family caregivers' decision process to institutionalize persons with Parkinson's disease: a grounded theory study. Int J Nurs Stud. (2012) 49:445–54. 10.1016/j.ijnurstu.2011.10.003 22036578

[5] Tan SB Williams AF Morris ME. Experiences of caregivers of people with Parkinson's disease in Singapore: a qualitative analysis. J Clin Nurs. (2012) 21:2235–46. 10.1111/j.1365-2702.2012.04146.x 22788558

[6] Mosley PE Moodie R Dissanayaka N. Caregiver burden in parkinson disease: a critical review of recent literature. J Geriatr Psychiatr Neurol. (2017) 30:235–52. 10.1177/0891988717720302 28743212

[7] Seppi K Ray Chaudhuri K Coelho M Fox SH Katzenschlager R Perez Lloret S. The collaborators of the Parkinson's disease update on non-motor symptoms study group on behalf of the movement disorders society evidence-based medicine committee, update on treatments for non-motor symptoms of Parkinson's disease-an evidence-based medicine review. Mov Disord. (2019) 34:180–98. 10.1002/mds.27602 30653247 PMC6916382

[8] Weintraub D Chiang C Kim HM Wilkinson J Marras C Stanislawski B. Antipsychotic use and physical morbidity in parkinson disease. Am J Geriatr Psychiatr. (2017) 25:697–705. 10.1016/j.jagp.2017.01.076 28259697

[9] Tomasino KN Lattie EG Ho J Palac HL Kaiser SM Mohr DC. Harnessing peer support in an online intervention for older adults with depression. Am J Geriatr Psychiatr. (2017) 25:1109–19. 10.1016/j.jagp.2017.04.015 28571785 PMC5600661

[10] Chien WT Clifton AV Zhao S Lui S Peer support for people with schizophrenia or other serious mental illness. Cochrane Database Syst Rev. (2019) 4:CD010880 10.1002/14651858.CD010880.pub2 30946482 PMC6448529

[11] de Wit J Vervoort SC van Eerden JM van den Berg LH Visser-Meily J Beelen MA. User perspectives on a psychosocial blended support program for partners of patients with amyotrophic lateral sclerosis and progressive muscular atrophy: a qualitative study. BMC Psychol. (2019) 7:35. 10.1186/s40359-019-0308-x 31202270 PMC6570885

[12] Smolensky L Amondikar N Crawford K Neu S Kopil CM Daeschler M. Fox Insight collects online, longitudinal patient-reported outcomes and genetic data on Parkinson's disease. Sci Data. (2020) 7:67. 10.1038/s41597-020-0401-2 32094335 PMC7039948

[13] Mantri S Klawson E Albert S Rapoport R Precht C Glancey S Carepartner communication on parkinson disease psychosis (4116). Neurology. (2020) 94 Available online at: https://n.neurology.org/content/94/15_Supplement/4749 (accessed December 28, 2020).

[14] Fredericks D Norton JC Atchison C Schoenhaus R Pill MW. Parkinson's disease and Parkinson's disease psychosis: a perspective on the challenges, treatments, and economic burden. Am J Manag Care. (2017) 23:S83–92. 28715903

[15] Berger S Chen T Eldridge J Thomas CA Habermann B Tickle-Degnen L. The self-management balancing act of spousal care partners in the case of Parkinson's disease. Disabil Rehabil. (2019) 41:887–95. 10.1080/09638288.2017.1413427 29228835 PMC6179928

[16] Feeney M Evers C Agpalo D Cone L Fleisher J Schroeder K. Utilizing patient advocates in Parkinson's disease: a proposed framework for patient engagement and the modern metrics that can determine its success. Health Expect. (2020) 23:722–30. 10.1111/hex.13064 32363785 PMC7495075

[17] Wetmore JB Li S Yan H Irfan M Rashid N Peng Y. Increases in institutionalization, healthcare resource utilization, and mortality risk associated with Parkinson disease psychosis: retrospective cohort study. Parkinsonism Relat Disord. (2019) 68:95–101. 10.1016/j.parkreldis.2019.10.018 31679990

[18] Chahine LM Chin I Caspell-Garcia C Standaert DG Brown E Smolensky L. Comparison of an online-only parkinson's disease research cohort to cohorts assessed in person. J Parkinsons Dis. (2020) 10:677–91. 10.3233/JPD-191808 31958097 PMC7242834

[19] Schneider RB Myers TL Daeschler M Tarolli C Adams J Barbano R Validation of fox insight cohort via virtual research visits (4749). Neurology. (2020) 94 Available online at: https://n.neurology.org/content/94/15_Supplement/4749 (accessed December 16, 2020).

[20] Balash Y Korczyn AD Migirov AA Gurevich T. Quality of life in Parkinson's disease: a gender-specific perspective. Acta Neurol Scand. (2019) 140:17–22. 10.1111/ane.13095 30953570

[21] Muller AJ Mills J O'Callaghan MZ Naismith C Clouston SL Lewis PD. Informant- and self-appraisals on the psychosis and hallucinations questionnaire (PsycH-Q) enhances detection of visual hallucinations in parkinson's disease. Mov Disord Clin Pract. (2018) 5:607–13. 10.1002/mdc3.12683 30637281 PMC6277359

